# A Pilot Study on the Effectiveness of Prazosin as a Treatment of Post-Traumatic Stress Disorder-Related Nightmares in Women with Bulimia Nervosa

**DOI:** 10.7759/cureus.42433

**Published:** 2023-07-25

**Authors:** Fauzia Mahr, Daniel A Waschbusch, Alexandros Vgontzas

**Affiliations:** 1 Pediatrics and Eating Disorders, Penn State College of Medicine, Hershey, USA; 2 Psychiatry and Behavioral Health, Penn State College of Medicine, Hershey, USA

**Keywords:** sleep, ptsd, bulimia, nightmares, prazosin

## Abstract

Objectives

Post-traumatic stress disorder (PTSD) symptoms are reported in over 36% of individuals with bulimia nervosa. To date, none of the clinical trials have examined nightmare reduction in this population. We evaluated the effectiveness of prazosin in bulimic females experiencing PTSD-related nightmares. We hypothesized that prazosin will decrease nightmares, normalize cortisol levels and secretory patterns, and improve sleep.

Methods

Our seven-week prospective, randomized, double-blind, placebo-controlled crossover pilot trial recruited eight adult women. Each participant received three weeks of prazosin and a placebo, separated by a one-week washout period. The order of treatment was counterbalanced across participants. Self-reports, clinician-administered scales, and salivary cortisol was collected to measure outcomes.

Results

A significant treatment effect was seen in nightmare intensity on the Clinician-Administered PTSD Scale (CAPS-I) (p=0.026) and a marginally significant effect on nightmare frequency (p=0.069). The only significant main effect of treatment on self-reported sleep parameters was on nightmares. Cortisol secretory patterns did not change, but on average, study participants had significantly higher cortisol levels compared to normative values. ANOVA showed a significant main effect of time for cortisol (F(4, 28) = 6.15, p=.001) but no within or between groups significant effects (ps>.179). Follow-up tests showed the effect of time was linear (F(1, 7) = 10.77, p=.013).

Conclusion

Prazosin significantly reduced intensity and marginally reduced the frequency of PTSD-related nightmares in bulimia nervosa but did not affect subjective sleep efficiency, quality, cortisol levels, or diurnal cortisol secretory pattern. Larger trials using objective sleep measures are warranted to replicate these findings.

## Introduction

Bulimia nervosa is characterized by recurrent episodes of binging followed by compensatory behaviors to purge the consumed food. This maladaptive behavior is driven by shape and weight concerns. Lifetime prevalence estimates are as high as 3%, with a higher prevalence in women [1]. Bulimia nervosa emerges later than anorexia nervosa and results in substantial psychosocial impairment throughout the illness [1,2]. 

Emotional dysregulation is a common behavioral manifestation of bulimia nervosa, and literature strongly supports a relationship between trauma and the development of bulimia nervosa. It has been hypothesized that trauma resulting in the development of post-traumatic stress disorder (PTSD) is a substantial risk factor for the later development of bulimia nervosa [3]. Moreover, purging behaviors seen in bulimia nervosa, such as self-induced vomiting, use of diet pills, and laxative abuse, have been reported as maladaptive strategies which may momentarily alleviate traumatic memories by decreasing the hyperarousal associated with PTSD, thereby minimizing trauma associations [4]. Conversely, about one-third (36.9%) of individuals with bulimia experience frequent symptoms of PTSD [5]. This includes nightmares, which are experienced by up to 80% of patients with PTSD [6, 7].

Multiple theories have been postulated regarding the neurobiological underpinnings of PTSD and associated nightmares. Excessive noradrenergic activity that does not decrease during sleep has been observed in PTSD and differentiates it from normal sleep [8]. Alpha-one (α1) adrenergic stimulation disrupts rapid eye movement (REM) sleep and increases stage one light sleep, thus increasing the likelihood of nightmares. Normal dreams arise during REM sleep, whereas trauma-related content is more likely to emerge during stage one light sleep. Reversing the noradrenergic hyperactivity seen in PTSD using an α1 adrenergic antagonist has been postulated to reverse REM disruption. This theory has been strengthened by studies that reported increased light sleep and REM disruption with methoxamine (an α1 agonist) challenge [9].

There are no FDA-recommended pharmacologic treatments specifically for nightmares in PTSD, but several clinical trials of prazosin have demonstrated efficacy in decreasing nightmares [7, 10, 11]. Prazosin is an α1 adrenergic antagonist at the postsynaptic receptors and is commonly used as an antihypertensive agent. It has also been used to alleviate nightmares stemming from PTSD [10]. Postsynaptic adrenergic α1 receptors are involved in fear responses and also modulate startle and sleep responses [8]. It has been hypothesized that nightmares stem from abnormal cortical arousal during sleep and that trauma increases the risk of cortical hyperarousal [6]. Reduction of nocturnal hyperarousal with prazosin may decrease nightmares by reversing REM disruption and decreasing stage one sleep [10].

Hyperarousal stemming from trauma activates the hypothalamic pituitary adrenal (HPA) axis leading to increased cortisol production to inhibit the stress response. However, chronic stress may serve to downregulate stress responses, leading to low cortisol levels [12, 13]. Studies reporting cortisol levels in PTSD have shown inconsistent results, likely secondary to variability in collection methods, collection time, and comorbidities. Low cortisol levels are associated with PTSD, but higher cortisol levels are associated with the co-occurrence of PTSD and major depressive disorder (MDD) [14]. Some researchers have reported reduced cortisol levels in abused women with bulimia nervosa [15], but other studies report higher cortisol levels in bulimia nervosa [16].

To date, none of the prazosin clinical trials in PTSD have been conducted in individuals struggling with bulimia nervosa, comorbid PTSD, and associated nightmares. In light of this gap in research, we conducted a pilot study to evaluate the effectiveness of prazosin in women with bulimia nervosa experiencing distressing nightmares secondary to PTSD. We hypothesized that prazosin administration would decrease nightmares and improve sleep. The secondary aim of our study was to assess cortisol levels and their secretory patterns in bulimic individuals with PTSD after prazosin administration. We hypothesized that prazosin would normalize cortisol levels and secretory patterns.

## Materials and methods

Participants

Ten participants were recruited from an eating disorders outpatient clinic. Two participants dropped out after initial consent and screening, resulting in a final sample of eight participants. The average age for the eight participants was 30.12 years, the average body mass index (BMI) was 22.75, and four (50%) had experienced sexual abuse. On average, the participants had experienced some form of trauma (emotional, physical, and /or sexual abuse) for a duration of 16.6 years. Inclusion criteria were: (a) female, (b) met the Diagnostic and Statistical Manual of Mental Disorders, Fourth Edition (DSM-IV) diagnostic criteria for bulimia nervosa and PTSD as diagnosed by a licensed psychiatrist, (c) between 18-45 years, and (d) evidence of nightmares on the Clinician-Administered PTSD Scale (CAPS) and the PTSD Checklist for Civilians (PCLC-C) with a score of three or higher on these rating scales. Exclusion criteria were: (a) presence of neurological, cardiac, or electrolyte abnormality that would impact the use of prazosin, (b) active narcolepsy, sleep apnea, or restless leg syndrome, (c) use of steroid or Beta blockers, and (d) alcohol or substance abuse within the past three months.

Procedures

The study was approved by an institutional review board. All participants provided informed written consent before participating in the study. The study used a randomized, double-blind, placebo-controlled, within-subjects (cross-over) design to compare the effects of prazosin to placebo in reducing nightmares in females with bulimia nervosa and PTSD. Each participant was enrolled for seven weeks, which included three weeks of receiving prazosin, three weeks of receiving a placebo (methylcellulose), and a one-week washout period between prazosin and placebo. The order of administration (prazosin versus placebo) was counter-balanced across participants. A one-week washout was determined based on previous data and the half-life of prazosin [8-10, 17-18].

Participants were evaluated by a psychiatrist at the start of their participation (initial visit) and once during each week of both the placebo and medication arms for a total of seven study visits. The same procedure was followed at each visit. Specifically, participants completed ratings to measure sleep, nightmares, and adverse events with the research assistant, then met with the psychiatrist to discuss these and their general functioning. Vital signs (BMI, EKG, comprehensive metabolic panel (CMP), liver function tests (LFTs), and urodynamic studies (UDS)) were also collected at each visit as well. During the medication weeks, the psychiatrist used clinical assessment and reports of adverse events or responses from the patient to adjust doses in 1-2 mg increments every seven days to achieve maximum therapeutic benefit. The pharmacy was distributing the medication or placebo, and the psychiatrist, research assistant, and patient were unaware of the medication vs placebo condition. Participants were contacted by the research assistant via phone the morning after dose initiation of either treatment arm (the research assistant was also unaware of treatment arms) and after each increased dose to monitor for possible adverse events. The maximum dose of prazosin allowed in the protocol was 6 mg daily, but the highest dose actually used during the study (based on participant-reported response and psychiatrist's assessment) was 3 mg daily.

There were no drug discontinuations during the study period, and medication was generally well tolerated. Headache and nausea were the most commonly reported side effects, which resolved within a couple of days. One participant presented to an emergency room due to feeling faint after a few days of taking prazosin but was discharged from the emergency room without medication changes.

Measures

Rating Scales

The Clinician-Administered PTSD Scale is a semi-structured interview and consists of 30 items. The frequency and intensity of specific symptoms are rated using a Likert response format that ranges from 0 (never) to 4 (daily or almost daily). A severity score for each symptom can be calculated by summing frequency and intensity scores. The frequency of nightmares was used as an inclusion criterion, with scores of 3 or 4 required for enrollment in the study [19].

The PTSD Checklist for Civilians consists of 17 items that are rated using a Likert response format that ranges from 1 (not at all) to 5 (extremely). Items can either be added up for a total score, or each response category can be looked at separately, with 3-5 considered moderate or above and 1-2 as asymptomatic or below moderate. One item on this scale assesses whether, over the past month, there have been repeated distressing dreams of a stressful event. In our study, a score of 3 or above was required for enrollment in the study [20].

The Pittsburg Sleep Quality Index [21] is a 19-item self-reported standardized questionnaire that assesses sleep quality over a one-month period and takes five to 10 minutes to complete. Items are rated using 0 to 3 scales that are summed to create seven scores that assess subjective sleep quality, sleep, sleep duration, habitual sleep efficiency, sleep disturbances, use of sleeping medication, and daytime dysfunction.

The Hamilton Depression Rating Scale [22] is a 17-item clinician-administered instrument to assess the severity of depression. Items are rated on either a 0 to 3 or a 0 to 5-point scale and are summed to compute a total score that ranges from 0 to 61.

The Sleep 50 Questionnaire [23] is a 50-item self-report questionnaire that assesses commonly presenting sleep problems. Five nightmares specific items from this scale were used in the study, and they were rated 1-4 on a Likert scale: "I have frightening dreams, I wake up from those dreams, I remember the content of the dreams, I can orient quickly afterward, and I have physical symptoms during or after dreams".

Cortisol

Cortisol samples were collected via saliva at three study points: at baseline, at the end placebo, and at the end of medication. At each of these points, five diurnal samples were collected (eight am, noon, three pm, six pm, and nine pm). Each participant chewed a piece of gum for over 60 seconds to stimulate saliva; a cortisol sample swab was given to participants for saliva collection. All saliva samples were frozen until the data collection ended. The saliva samples were spun by trained professionals in an assay in a laboratory. All cortisol samples were collected at the same time points to allow for accurate comparisons.

Data analyses

Effects of prazosin medication were examined by computing a series of one-way repeated measures analyses of variance (ANOVAs), with treatment (baseline vs. placebo vs. medication) as the independent variable and measures of nightmares, sleep, and bulimia as the dependent variables. Significant ANOVAs were followed up by comparing treatment conditions in post hoc t-tests with Holm's adjusted p-values and by computing Cohen's D effect sizes. Cortisol was examined by computing a 3 (treatment: baseline vs. placebo vs. medication) x 5 (time: eight am vs. noon vs. three pm vs. six pm vs. nine pm) repeated measures ANOVA, with simple effects tests and post hoc t-tests to follow up significant results. Analyses were computed in JASP version 0.16 [24].

## Results

Primary outcome

Nightmares

Results of the repeated measures ANOVAs (Table 1) showed a significant effect of treatment for the CAPS-I (nightmare intensity), the Sleep 50 questionnaire nightmares item, and a marginal effect for the CAPS-F (nightmare frequency) score. These results (Table 1) showed that nightmare scores were lower on medication than during baseline. Medication did not differ from placebo on any other measure, and baseline and placebo differed only for the Sleep50 measure.

**Table 1:** Means, standard deviations, one-way ANOVA results, and effect sizes of sleep parameters CAPS = Clinician-Administered PTSD Scale, Freq = frequency, PCLC = PTSD Checklist for Civilians, PSQ = Pittsburg Sleep Quality Index, B = baseline, P = placebo; M = medication. Post hoc tests show groups that differed at p<.05 (Holm's adjusted), except where the group is indicated by a+, which indicates that groups differed at p<.10 (Holm's adjusted).

Measure	Baseline		Placebo		Medication		ANOVA results			Cohen’s D effect sizes		
	Mean	SD	Mean	SD	Mean	SD	F-value	p	Post-hoc	B vs. P	B vs. M	P vs. M
Nightmares												
Sleep-50	3.62	0.52	2.54	0.73	2.58	0.71	6.15	0.012	B > P B > M	1.09	1.05	-0.04
CAPS-Intensity	3.62	0.74	2.75	1.28	1.75	1.28	4.79	0.026	B > M	0.51	1.09	0.58
CAPS-Freq	3.63	0.52	2.75	1.28	1.88	1.55	3.25	0.069	B > M^+^	0.45	0.9	0.45
PCLC Dreams	4.5	0.53	3.37	1.06	3.5	0.76	4.02	0.042	B > P^+^ B > M^+^	0.92	0.81	-0.1
Sleep												
PSQ latency	2.75	0.46	2.25	0.71	2.5	0.54	1.61	0.234	No diffs	0.64	0.32	-0.32
PSQ duration	2.63	0.52	2.37	0.52	2.37	0.52	1	0.393	No diffs	0.43	0.43	0
PSQ disturbance	1.87	0.84	1.87	0.35	1.75	0.89	0.15	0.863	No diffs	0	0.17	0.17
PSQ quality	0.25	0.46	1.13	0.99	0.88	0.64	2.81	0.094	No diffs	-0.81	-0.58	0.23

Secondary outcomes

Sleep

Results of repeated measures ANOVAs (Table 1) showed no significant differences for the PSQ sleep subscales. Prazosin did not lead to statistically significant improvements in sleep efficiency, sleep quality, sleep latency, or sleep duration.

Cortisol

**Figure 1:**
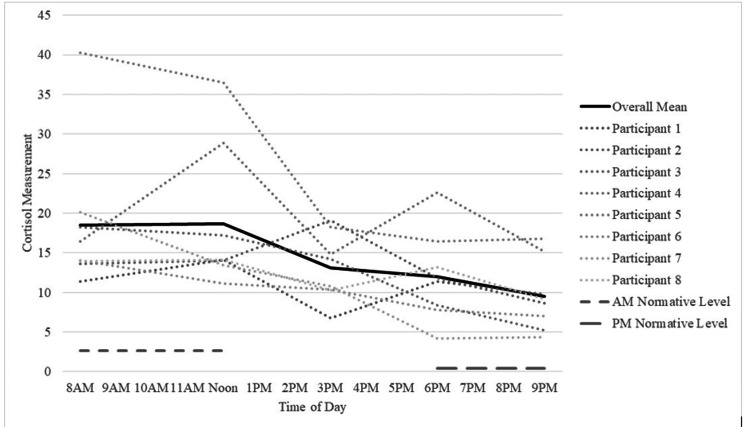
Cortisol levels for the overall sample and for each participant Illustration of cortisol levels over time of day. The group mean is shown as a solid black line. Individual plots for each of the eight participants are shown as dotted lines. Normative values of cortisol are shown in the lines with short dashes (am norms) or long dashes (pm norms).

Figure 1 shows the diurnal cortisol rhythm, as measured using diurnal cortisol slope (DCS). The average across participants and the average levels of cortisol in the general population are also illustrated. As shown, cortisol levels were higher in study participants compared to normative values and followed normal secretory patterns of higher levels upon awakening and then a steady decline during the day. Specifically, the eight am cortisol value averaged across all participants (18.51) significantly differed from the normative am value (2.27). t (7) = 6.68, p = .0003, Cohen's D effect size = 2.36. Similarly, the nine pm cortisol value averaged across all participants (9.52) significantly differed from the normative pm value (0.40). t (7) = 3.75, p=.0072, Cohen's D effect size = 1.33. These results show that, on average, the study participants had significantly higher cortisol levels compared to the normative cortisol levels at both time points (eight am and nine pm values were used for comparison because only these reference time points were available in the cortisol analysis kit).

**Figure 2:**
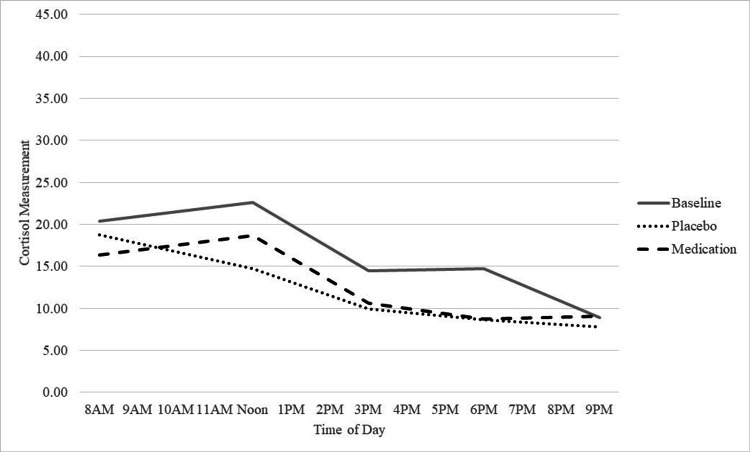
Cortisol levels separately for each treatment arm Illustration of cortisol levels over time of day separately for each treatment condition

Effects of treatment and time on cortisol were evaluated using a 3 (treatment) x 5 (Time) repeated measures ANOVA. Results showed a significant main effect of time (F (4, 28) = 6.15, p=.001) but no other significant effects (ps > .179). Follow-up tests showed the effect of Time was linear (F (1, 7) = 10.77, p=.013) showing that cortisol decreased over the day. For descriptive purposes, Table 2 show means, standard deviations, and effect sizes comparing cortisol as a function of time of day and treatment condition, and Figure 2 shows cortisol levels over the course of the day separately for each treatment condition. As shown, there were no notable differences between medication and placebo.

**Table 2:** Means, Standard Deviations, and Standardized Mean Difference Effect Sizes for Cortisol

Means (SD)	Time of day					
	8:00 am	Noon	3:00 pm	6:00 pm	9:00 pm	Overall
Baseline	20.4 (13.2)	22.6 (17.0)	16.2 (8.5)	16.5 (14.5)	9.9 (6.4)	17.1 (12.7)
Placebo	18.8 (13.4)	14.7 (7.6)	11.1 (6.2)	9.6 (5.9)	8.6 (7.7)	12.6 (9.0)
Medication	16.3 (5.4)	18.7 (10.6)	11.9 (6.1)	9.7 (5.9)	10.1 (6.8)	12.6 (7.7)
Effect sizes						
Baseline vs. placebo	0.16	0.78	0.5	0.68	0.13	0.45
Baseline vs. medication	0.4	0.39	0.42	0.67	-0.02	0.45
Placebo vs. medication	0.24	-0.39	-0.08	-0.01	-0.15	0

## Discussion

It is well established that nightmares are commonly associated with both PTSD and bulimia nervosa. It is hypothesized that prazosin, an α1 adrenergic antagonist, may be an effective medication for addressing nightmares in PTSD, but this hypothesis has not previously been tested in bulimia nervosa and comorbid PTSD with nightmares. Our pilot study took an initial step toward this goal by using a seven-week randomized placebo-controlled trial that examined the effects of prazosin on nightmares in women diagnosed with active bulimia nervosa symptoms and PTSD. Results (Table 1) showed that prazosin produced a reduction in the intensity of nightmares, supporting our primary hypothesis. However, prazosin did not change other sleep parameters (quality, efficiency) or cortisol levels and its secretory pattern (Figure 1). 

Despite using a small sample size, results from our double-blind placebo-controlled study found that prazosin significantly reduced the intensity of PTSD-related nightmares as measured by CAPS and marginally reduced their frequency as well. A similar decrease in nightmares was also observed in the self-report of a nightmare item on the Sleep 50 questionnaire. Our results concur with previously published data supporting the effectiveness of prazosin in PTSD [8, 10-11, 25] but extend these findings to suggest that the same effect may occur in women with bulimia and PTSD. It is noteworthy that a previous large multicenter study failed to show the benefits of this alpha-1 adrenergic blocker in veterans with chronic PTSD. In their trial, prazosin failed to show an appreciable decrease in nightmares or improvement in sleep quality [18]. Notably, our population consisted of civilian women, and there was a difference in comorbidities between the studies. In our pilot study, although participants reported a decrease in nightmares with prazosin, it failed to demonstrate any significant positive impact on other sleep parameters, including sleep efficiency, sleep quality, sleep latency, or sleep duration. (Table 1). It is possible that prazosin primarily decreases nightmare intensity in bulimic patients with PTSD and does not render discernible benefit for other sleep parameters in bulimia nervosa and comorbid PTSD.

We had also hypothesized that prazosin would impact cortisol levels and affect cortisol diurnal secretory patterns. The normal secretory rhythm of cortisol secretion reflects low cortisol upon awakening, which peaks within 30-40 minutes and then steadily declines during the rest of the day [12, 26]. Current evidence suggests that stress affects the HPA axis, and chronic stress may downregulate the HPA axis leading to abnormalities in cortisol secretion [12-13]. The cortisol levels in our sample (Figure 1) were significantly higher than in typical adult normative values (i.e., adults without PTSD or bulimia). This is consistent with several existing research studies, which also found high cortisol levels in women with bulimia nervosa [16, 27]. Malnutrition is also reported to impair stress response [28], and stressful events increase cortisol levels and lead to urges to binge [16]. However, some researchers have reported low cortisol levels in abused women with bulimia nervosa [29-30]. Thus, research on cortisol levels in bulimia nervosa is inconsistent, perhaps due to secondary factors such as comorbidities, nutritional status variability, variations in duration of trauma experience, and lack of uniformity in cortisol sample collection methods.

The diurnal cortisol slope (DCS) (Table 2, Figure 1) showed the expected change over the course of the day, with higher levels in the morning and lower levels later in the day. In fact, there is evidence that time of day accounts for 72% of the difference in diurnal salivary cortisol levels [26]. More central to this study, the cortisol pattern did not differ as a function of treatment conditions (baseline, placebo, or medication). In other words, our sample had normal cortisol secretory pattern at baseline, and it did not change with medication. This finding concurs with literature reporting circadian variation maintenance in normal-weight bulimia nervosa [27], suggesting that the same pattern is apparent in women with bulimia, PTSD, and above-average body mass index.

Although there were important strengths to this study, including the use of a within-subjects design and the use of a placebo condition, several limitations must also be acknowledged. First and most important, is that the study had a small sample size. This is appropriate for a pilot study, and our primary hypothesis was supported, but the results of significance tests were underpowered and should be considered alongside estimates of effect sizes. Second, we did not conduct objective screening for sleep apnea or collect other objective sleep measurements. This is important because undiagnosed apnea may limit the benefits of prazosin by interfering with it. However, all subjective measures used in this study were valid and reliable. Third, in our study, prazosin was only administered as nighttime doses to avoid hypotension often noted in bulimia nervosa, which could have limited its efficacy compared to twice daily dosing. Despite using only once-daily dosing, prazosin showed promising results in reducing nightmares. Lastly, the presence of comorbidities likely impacted the subjective reports on the validated measures.

## Conclusions

Despite these limitations, our pilot study demonstrated the effectiveness of prazosin for women with bulimia nervosa and PTSD. To our knowledge, this is the first study reporting on the effectiveness of prazosin in bulimic participants experiencing PTSD-related nightmares and provides evidence that prazosin decreased the intensity and frequency of nightmares in this seven-week pilot trial. These results, though preliminary due to the small sample size, provide early evidence in support of the use of this alpha-1 adrenergic blocker in bulimia nervosa. The medication was well tolerated and merited further investigation using objective sleep polysomnographic measures and a larger sample size for extensive validation. Considering the high prevalence of PTSD with bulimia nervosa, the combined effects of cognitive behavioral therapy (CBT) and prazosin on PTSD-related nightmares are also worth investigating in bulimia nervosa. 
